# Assessing Validity and Bias in a Peer Evaluation Tool: Associations with Demographics and Performance in Team-based Learning

**DOI:** 10.1007/s40670-026-02759-8

**Published:** 2026-05-20

**Authors:** Kyle A. Robertson, Elizabeth R. Agosto, Jessica N. Byram

**Affiliations:** Indiana University School of Medicine, 715 Clinic Dr., West Lafayette, Indianapolis, IN 47907 USA

**Keywords:** Peer evaluation, Team-based learning, Preparation, Bias

## Abstract

A formative Peer Evaluation Tool (PET) was developed for use in a Team Based Learning (TBL) graduate histology course. The PET has been evaluated for content and construct validity evidence with three dimensions that assess preparation, providing feedback, and feedback receptivity and attitude. The aim of this work is to evaluate further validity evidence by analyzing the relationship between PET scores, demographic variables, individual readiness assessment test (iRAT) scores, and the preparation dimension of the PET for five cohorts of students. Differences between timepoints (e.g., PET1 vs. PET2) were assessed via paired t-tests. Spearman’s correlation and linear regression analyses were performed to investigate the relationship between all factors (SPSS v.29). There is a significant (*p* < 0.05) difference between timepoints for all factors except iRAT scores. There is a significant positive correlation among all scores, except between Prep1&2 and TBL1&2 scores with the strongest correlations between PET1&2 and Prep1&2 scores. PET scores have no correlation to demographics. Preparedness to the team is critical for TBL and these results suggest that Prep scores can predict preparation for iRATs, suggesting that students can evaluate their peers’ preparedness for TBL. This study provides additional evidence for the value and use of this formative PET.

**Supplementary Information:**

The online version contains supplementary material available at https://doi.org/10.1007/s40670-026-02759-8.

## Introduction

Peer evaluation is an important aspect of collaborative learning as it gives students opportunities to provide and receive feedback on specific criteria related to team learning [1]. Within the team-based learning (TBL) pedagogy, these assessments can be formative or summative, and a variety of methods have been proposed [2–4]. Specifically, “formative (process) information” is thought to inform students on their performance over time, is low stakes or non-graded, and thus promotes the development of interpersonal and team skills in real time [3]. Comparatively, the “summative (outcome) data” is thought to provide the instructor with a means to “ensure fairness in grading” [3]. Li et al., [4] explored factors influencing the effect of peer assessment and found the “effect size for both scores and comments were similar, whereas the effect size for comments only was smaller” [4], thus, highlighting the need for aspects of both summative and formative assessment in the peer evaluation process.

Despite the positive effects of peer assessment on learning outcomes [4] and motivation, concerns about the accuracy and validity of peer assessments persist [5]. Particularly, of concern to both students’ receiving peer evaluation and instructors who implement peer evaluation in their team-based learning classrooms are biases in the evaluations based on sex and/or gender, ethnicity, and team dynamics [6].

Reviews of bias in peer assessment demonstrate mixed evidence regarding differences in evaluations by sex and/or gender with no consistent pattern based on who gives or receives more favorable evaluations. Specifically, when women receive higher peer ratings, these differences often parallel higher course grades [7, 8]. For example, an analysis of more than 8,000 peer evaluations in a team-based learning context over 13 years found that women earned higher ratings than men in introductory and intermediate course, but this did not hold true in advanced courses [8]. Similarly, studies that reported lower peer ratings for males also found them to have lower grade point averages (GPA) and thus suggest this “could be a function of lower ability or performance rather than gender bias [9]. Evidence regarding racial and ethnic bias is likewise inconsistent, with some studies finding no bias and other reporting lower ratings for students of color compared to their white peers [7] Notably, a greater proportion of students reported experiencing bias from peers in their small groups compared to those reporting perceiving bias in their peer assessments [6].

When considering the method of peer evaluation, summative methods may be more susceptible to bias than formative assessment methods. Some forms of peer evaluation require assigning points (e.g., balance method, Fink Method), where a set number of points are to be distributed to peers, with some instructors requiring differing point values per peer [2]. When using the balance method, Stonewall and colleagues [6] found biases in score distributions across race/ethnicity, gender, international versus domestic students, and English as a second language learners that are independent of GPA. Furthermore, a friendship bias phenomenon may result from team and personal dynamics where some students may be more inclined to give higher points to peers who are friends [6, 10].

Through the framework of mitigating and acknowledging the existence of bias, there is a continued need to have reliable and valid instruments for peer evaluation for use in the TBL environment [11]. In our previous work [12], we describe the development of our peer evaluation tool (PET) utilized in a graduate histology course that employs TBL pedagogy. As one of the cornerstones of a TBL curriculum, the PET is a means for students to assess their peers and self on their contributions to team performance and identify opportunities for improvement. The evidence gathered for internal structure and reliability of the PET was investigated through exploratory factor analysis (EFA), confirmatory factor analysis (CFA), and Cronbach’s alpha using data from four cohorts (2019–2022). The results of both the EFA and CFA demonstrated a three-factor structure with a good to very good fit for the dimensions of Preparation, Providing feedback, and Feedback receptivity and attitude.

However, additional evidence of validity is necessary as “validity is never assumed and is an ongoing process of hypothesis generation, data collection and testing, critical evaluation and logical inference” [13]. Additionally, our initial investigation was not without limitations, such as exploring the effects of student demographics to investigate response biases, students’ perception of the peer evaluation process, and the lack relation to other variables (i.e., convergent, and divergent) [11, 12, 14]. Building upon our previous work, in this study we continue to construct a narrative by providing further supporting evidence of validity by exploring the relationship of the PET to other variables to promote the use of the PET in the TBL environment [12].

Furthermore, we prescribe to the concepts that “validity” is not an all or none proposition, but instead as based on the S*tandards for Educational and Psychological Testing* it “is the degree to which all the accumulated evidence supports the intended interpretation of test scores for the proposed use” [15]. Henceforth, aligning with the unitary perspective of validity, the *Standards* propose five sources of validity evidence, based on Messick [16] with not all sources required in every setting: (1) “Test content,” (2) “Response Processes,” (3) “Internal structure,” (4) “Relation to other variables,” and (5) “Consequences” [15]. Thus, validity is conceptualized as a unitary concept, with all validity being construct validity and is further supported from multiple sources of evidence as set forth by the *Standards* [13, 15, 17–19].

As our first investigation demonstrated the PET to have evidence of construct and internal structure validity, this follow-up study will explore validity evidence based on relations to other variables. Therefore, we seek to understand the relationship between Preparation scores, PET scores, TBL team and individual scores, and individual Readiness Assessment Tests (iRATs). We hypothesize that Preparation scores on the PET will be predictive of iRATs as they are intended to assess students’ advanced preparation prior to the TBL session. In addition to our assessment of criterion validity, we will further assess whether any differences exist in PET scores based on demographics (i.e., self-reported gender and ethnicity) to assess any potential bias these variables may have on aggregate peer evaluation scores. We hypothesize that our formative PET will be resistant to group level biases via relations of PET (aggregated group scores) to gender and ethnicity, common in summative peer evaluation methods.

## Method

### Study Context

This study took place in the context of a graduate histology course for students enrolled primarily in pre-professional Master of Science programs and doctoral programs in biomedical research. The course is designed as a traditional TBL course that includes the seven core design elements outlined by [20]. Students are assigned to teams of 5–7 students and remain with their group for the entirety of the semester. Students are assigned to teams in a semi-random fashion where they were initially divided by degree program as it is thought that students in varied degree programs may have different experiences which may be of extrinsic value to their team.

Students take part a total of 10 TBL sessions across the semester and each TBL begins with a 10-question multiple choice iRAT to assess the students’ level of advanced preparation for the session. The same 10-question quiz is then taken with the TBL group (group readiness assurance test [gRAT]), but teams have two opportunities to answer a question correctly and can earn half credit on the second attempt. Lastly, students take part in an 8-question multiple choice application exercise with their teams that consists of higher order functional histology and case-based clinical questions. It is during these group exercises where students can evaluate how well their peers prepared for the session by their ability to thoughtfully contribute their knowledge to answering questions.

Each iRAT is worth 10 points, the gRAT is worth five points, and the application exercise is worth 15 points. As such, each TBL is worth 30 points, for a total of 300 TBL points across the semester. Group scores for each TBL session (averaged iRAT, gRAT, and application exercise score) are calculated and added across the semester as the groups compete for an incentive to be chosen by the winning team. All scores are aggregated with no score range, outliers, or other means of identifying individual student scores. Further, the gRAT and application exercises are collaborative efforts where all team members receive the same score. Team rankings are presented to students following each TBL session.

The PET includes 10 items rated on a four-point Likert-scale from never to always (0–3; maximum score of 30). Students complete the PET at the midpoint (PET1) and end of the semester when it is thought that they have sufficient evidence to evaluate their peers on the dimensions of the tool: preparation for TBL, ability to give feedback, and their receptivity to feedback receptivity and attitude about group work (PET2; after TBL5 and TBL10, respectively). Students earn points for thoughtful completion of the PET, for a total of 1% of their final grade, but not for the scores received on the instrument. Additional details about the PET and course structure can be found in Robertson et al., 2024.

### Data Collection

Data were collected across five cohorts of students (2020–2024) and included: PET1, PET2, averaged iRAT scores at midpoint (iRAT1) and end-of-semester (iRAT2) as well as scores of the preparation dimension (Prep1 and Prep2; comprised of three items from the PET, maximum score of nine), TBL team scores at midpoint (TBL1) and end of semester (TBL2), and the total points earned from TBL (TBLT) as an individual. Demographics were collected in a pre-course survey and include self-reported gender (woman; man; transgender; non-binary, genderqueer, gender fluid; prefer not to disclose; another identity not listed) and race/ethnicity (Asian (Asian; Middle Eastern; or Native American/Pacific Islander); Black African American; Hispanic, Native American; White). Specifically, the 3 items comprising the preparation dimension (Prep1 and Prep2) are:

1. “is well prepared for team activities.”
2. “shows the appropriate depth of knowledge to make a positive contribution to the team.”
3. “strives to learn more than the bare minimum necessary to complete the activity.”

These preparation items measure how a team member perceives their peer’s preparation prior to attending the in-person TBL session based on their interactions with their peers in the TBL environment, specifically when working together to answer gRAT and group application questions.

### Data Analysis

Descriptive statistics were performed on both the demographic data and all factors (PET1&2, iRAT1&2, Prep1&2, TBL1&2, and TBLT). Differences between the midpoint and end of semester scores for PET, iRAT, Prep, and TBL (e.g. PET1 vs. PET2) were assessed using paired t-tests.

All factors (PET1&2, iRAT1&2, Prep1&2, TBL1&2, and TBLT) were tested for normality using a Shapiro-Wilk test (*p* ≤ 0.05). All factors except TBLT are normally distributed; therefore, non-parametric analyses were performed. Relationships among all factors were assessed using Spearman’s correlation. Additionally, Spearman’s correlation was used to assess the relationship between PET scores and ethnicity, and the relationship between PET scores and gender were assessed using a point biserial Spearman correlation due to the dichotomous nature of the self-reported genders. Four factors (PET, iRAT, Prep, and TBL team scores) represent scores taken at different points during the course. Differences between these measures were analyzed using a paired t-test to assess any changes over time. Statistical significance determined by *p* ≤ 0.05.

Multiple linear regression analyses were performed to assess the predictive relationship between PET scores and all other factors (Prep, iRAT, TBL, and TBLT scores), and between iRAT scores and all other factors (PET, Prep, TBL, TBLT scores; *p* ≤ 0.05). Each model was bootstrapped with 1000 iterations. Shapiro-Wilk tests were performed on the residuals of each model to assess normality; residuals of all multiple linear regression models follow a normal distribution. Statistical significance determined by *p* ≤ 0.05. All analyses performed using IBM SPSS Statistics (version 29) and R version 4.5.1 [21].

This study received exempt status from the Institutional Review Board at [anonymized] (IRB #2012257742).

## Results

The five cohorts represented in our sample have a combined total of 205 students, with the 2020 cohort having the least number of students (32; 15.6%) and the 2021 cohort having the most (48; 23.4%) with further cohorts: 2022 (39; 19%); 2023 (40; 19.5); 2024 (46; 22.5%). Most students are women (135; 66%), and identify as white (91; 44.6%), followed by Black/African American (54; 26.5%), Asian (38; 18.6%; includes individuals who reported ethnicities of Asian, Middle Eastern, and Native Hawaiian/Pacific Islander), Hispanic (21; 10.3%), and Native American (1; 0.01%, a single individual). Missing data for PET1, PET2, and incomplete iRAT data were removed; however, only 1–2 datum were missing per cohort. There was no missing demographic data. See Table S1 in the Supplementary Materials for full demographic breakdown between cohorts.

Average scores for all factors, both for the total sample and each cohort, can be found in Table 2. Paired t-tests show significant differences between PET1&2, TBL1&2, and Prep1&2 scores. However, differences between iRAT1&2 scores are not significantly different (see Table 1).

**Table 1:** Average PET, Prep, iRAT, and TBL scores*. Statistically significant p-values are bold

	Average	Cohort					
	All	2020	2021	2022	2023	2024	*p*-value
PET							
PET1	25.74	27.10	25.34	25.05	24.84	26.57	**< 0.001**
PET2	26.75	27.06	25.64	26.69	26.79	27.7	
Prep							
Prep1	7.87	8.37	7.80	7.81	7.49	7.96	**< 0.001**
Prep2	8.1	8.11	7.88	8.21	7.97	8.33	
iRAT							
iRAT1	0.76	0.81	0.79	0.78	0.70	0.74	0.191
iRAT2	0.77	0.80	0.78	0.79	0.68	0.81	
TBL Team							
TBL1	132.52	133.64	130.99	136.75	130.73	131.34	**< 0.001**
TBL2	133.78	132.99	131.15	137.39	131.04	136.4	
TBL Individual							
TBLT	266.41	266.66	262.26	274.35	261.77	267.87	-

* Max PET score = 30; Max Prep score = 10; iRAT scores are percentages; Max TBL Team score = 150; Max TBL Individual score = 300

### Correlation Analyses

**Fig. 1:**
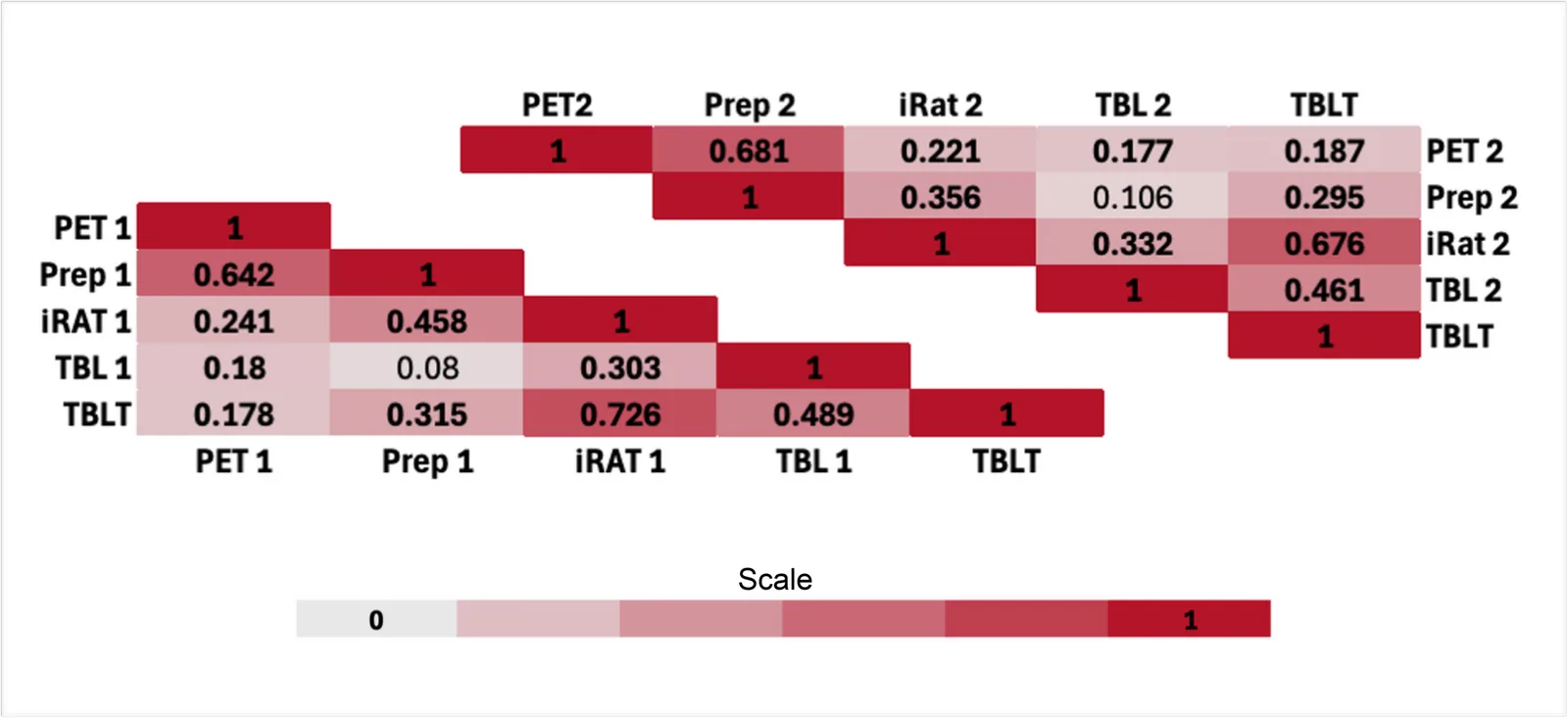
Heat map depicting strength of correlations among all study factors by timepoint; lower triangle is mid-semester (1) and upper triangle is end of semester (2). Darker colors indicate stronger correlation among factors; bold values are statistically significant

PET scores do not have a significant relationship with either gender (PET1: *r* = 0.006, *p* = 0.927; PET2; *r* = -0.063, *p* = 0.37) or ethnicity (PET1: *r* = 0.096, *p* = 0.173; PET2; *r* = 0.084, *p* = 0.231). Correlation coefficients show a very weak association (0.0≤ *r* ≥ 0.2) between PET1 and both TBL1 and TBLT, Prep1 and TBL1, PET2 and TBL2, and Prep2 and TBL2; a weak association (0.4 ≤ *r* > 0.2) between iRAT1 and both PET1 and TBL1, Prep1 and TBLT, PET2 and both iRAT2 and TBLT, Prep2 and both iRAT2 and TBLT, and between iRAT2 and TBL2; a moderate association (0.6 ≤ *r* > 0.4) between Prep1 and iRAT1, and between TBLT and both TBL1&2; and a strong association (0.8 ≤ *r* > 0.6) between PET1 and Prep1, iRAT1 and TBLT, PET2 and Prep2, and between iRAT2 and TBLT. Correlations among all factors are statistically significant, except for those between Prep1&2 and TBL1&2 scores. See Fig. 1 for a heatmap depicting the strength of the relationships among all factors within each time period.

### Multiple Linear Regression Analyses

Multiple linear regression analyses were performed to assess which factors best explain the variance in the PET and iRAT scores and could be used as predictors for these measures. Of the factors included in the model (Prep1, iRAT1, TBL1, and TBLT), only Prep1 is a significant predictor of PET1 scores, and explains approximately 51% of the variance observed in PET1 scores (adjusted R^2^ [aR^2^] = 0.507; *p* < 0.001; see Table 2). Whereas approximately 61% of the variance in PET2 scores can be explained by both Prep2 and TBL2 scores (aR^2^ = 0.606; *p* < 0.001; see Table 2), with iRAT2 and TBLT scores not being predictive factors. Models for both iRAT1 and iRAT2 scores show that these measures can be predicted by both the corresponding Prep scores (Prep1&2, respectively) and TBLT scores, with these factors explaining approximately 60% of the variance in iRat1 scores (aR^2^ = 0.602; *p* < 0.001), and approximately 50% of the variance in iRAT2 scores (aR^2^ = 0.498; *p* < 0.001; see Table 2). Neither PET scores nor group TBL scores are predictive of iRATscores.

**Table 2:** Multiple regression models predicting PET1, PET2, iRAT1, and iRAT2 scores^a^

Dependent Variable: PET1						Dependent Variable: PET2					
Predictors	B	CI		t	*p*-value	Predictors	B	CI		t	*p*-value
(Intercept)	8.78	1.36–16.47		2.75	**0.007**	(Intercept)	6.65	0.45–12.81		2.52	**0.013**
Prep1	1.82	1.45–2.15		13.85	**< 0.001**	Prep2	1.97	1.59–2.35		16.89	**< 0.001**
iRAT1	-1.58	-4.59–1.75		-1.28	0.203	iRAT2	-0.76	-2.77–1.24		-0.78	0.436
TBL1	0.04	-0.02–0.10		1.69	0.093	TBL2	0.07	0.021–0.12		3.33	**0.001**
TBLT	-0.01	-0.03–0.02		-0.44	0.662	TBLT	-0.02	-0.04–0.01		-1.65	0.101
Observations	205					Observations	205				
R^2^ / R^2^ adjusted	0.516 / 0.507	F = 53.375			**< 0.001**	R^2^ / R^2^ adjusted	0.613 / 0.606	F = 79.342			**< 0.001**
Dependent Variable: iRAT1						Dependent Variable: iRAT2					
Predictors	B	CI		t	*p*-value	Predictors	B	CI		t	*p*-value
(Intercept)	-1.042	-1.4 – -0.667		-6.124	**< 0.001**	(Intercept)	-1.019	-1.4 – -0.667		-5.664	**< 0.001**
PET1	-0.005	-0.015–0.005		-1.276	0.203	PET2	-0.004	-0.014–0.008		-0.781	0.436
Prep1	0.043	0.022–0.068		4.318	**< 0.001**	Prep2	0.037	0.012–0.061		2.899	**0.004**
TBL1	-0.002	-0.005–0.001		-1.378	0.170	TBL2	0.001	-0.002–0.004		0.472	0.638
TBLT	0.007	0.006–0.008		13.394	**< 0.001**	TBLT	0.006	0.004–0.007		10.483	**< 0.001**
Observations	205					Observations	205				
R^2^ / R^2^ adjusted	0.609 / 0.602	F = 77.981			**< 0.001**	R^2^ / R^2^ adjusted	0.507 / 0.498	F = 51.506			**< 0.001**

^a^ Abbreviations: *B* unstandardized coefficient, *CI* 95% Confidence Interval, *t* t value, *R*^*2*^  R Square, *F* F statistic

## Discussion

This study demonstrates additional validity evidence to support the use of the PET in TBL classrooms. PET was designed as a measure to assess peers in a team-based setting, to evaluate their preparedness for the session, their contributions to team performance, and to provide formative feedback to identify opportunities for improvement. The relationship, explored through multiple regression analysis, between the PET and Prep scores and the iRATs partly explains that students can adequately evaluate their peers’ preparedness for the session and as such the instrument is measuring one aspect of team performance in a TBL setting. Furthermore, significant differences in the PET and Prep scores between the two timepoints may indicate that students are considering the formative feedback from the instrument to modify (e.g., improve) their teaming performance in the second half of the course. However, future investigations would be needed to confirm whether students changed their approach to TBLs based on the peer evaluation process.

Few others have evaluated the validity evidence for peer evaluation instruments for use in TBL settings. Kweon and Park [22] developed a peer evaluation scale in a TBL course (PES-TBL) and evaluated the tool for evidence in construct, convergent and discriminant validity utilizing standard regression coefficient and average variance extracted, maximum share variance, and reliability coefficient. However, these sources of validity evidence are exploring constructs within the same instrument. Similarly, Fete and colleagues [10] assessed their peer evaluation instrument for evidence of content and structural validity, whereas in our current study we provide validity evidence for the PET with relation to external variables (e.g., TBLT, TBL1&2, iRAT1, and iRAT2). Using the balance method with discriminating points, Levine and colleagues [2] found moderate correlations between mean peer evaluation scores and knowledge-based variables (iRATs and board exam scores) which suggest that students value teammates with strong cognitive abilities. Notably, our formative PET provides students with feedback on other interpersonal and professionalism skills that are nonetheless important in the TBL setting [23].

An important finding was that there were no differences in PET scores received across race/ethnicity or gender which indicates ratings are unlikely to be biased by those demographics. While teams have purposive diversity based on degree programs, they are otherwise variable based on gender and race/ethnicity. Considering the predominance of women in the course (Table S1) there are instances when teams may be entirely women but have diversity in race/ethnicity. Regardless, this is promising as peer assessments have shown varying degrees of bias by sex/gender and race/ethnicity of the scores received by the students who are in minoritized groups [6, 7]. While this study found no differences in aggregate scores received by gender and race/ethnicity, we did not evaluate whether there were differences in the scores ***given*** by these same factors and whether differences exist at the level of the individual evaluator. Considering that each PET score is an aggregate of typically five evaluations from separate peers, this is likely protective of any individual biases that may occur. Furthermore, with a weak correlation demonstrated between TBL2 (TBL team score at end of semester) and PET2, this indicates the possibility of minimal subjective influence based on the competitive nature of the scoring system. However, further explicit investigation into how students perceive the competitive nature and how that may influence peer evaluation will need to be further explored.

TBL team scores were included in the analysis as a proxy to measure the influence of team dynamics on evaluation. One could hypothesize that teams who are scoring highly on TBL activities may be more likely to rate each other more favorable than teams who are lower in the team ranking. One concern regarding the validity of peer assessments, particularly in summative evaluations, are the influences of friendships and group dynamics, and the ability to “game the system” by giving high marks to friends [6, 10]. Our results found no relationship between PET or Prep scores by TBL team total which indicates students in high performing teams are giving their peers more favorable peer evaluations.

In our previous work, we provided several suggestions for implementation of our PET that continue to be supported by this study [12]. We suggest a formal introduction to the PET, including an overview of the purpose of the instrument and an opportunity to practice using the tool to provide peer feedback. There is a net positive effect when students are trained on peer evaluation instrument, understand the purpose of peer evaluation, and have transparency in the process [4, 5]. In a meta-analysis evaluating the effects of peer assessment on learning outcomes, Li and colleagues [4] found rater training and computer-mediated assessments to have the largest effect on learning gains. Implementing the PET in a digital format facilitates completion and allows for easy aggregation of team ratings such that individual evaluations are anonymous to the receiver.

Our study further supports the use of formative, criterion-based peer evaluation over summative methods. Some students are reluctant to give points that may negatively impact the grades of their peers [10] and forcing unequal point distributions for peers may exacerbate biases present in team-based settings [6, 24]. Therefore, the use of summative peer evaluation methods may be perceived as detrimental to overall student performance, team dynamics, and moreover may inadvertently introduce biases into the peer evaluation process.

## Limitations

This study is limited in generalizability due to it taking place in the context of a single course at one institution. It is unclear whether these results would be replicated if the PET were implemented in another TBL course or if the tool would be applicable across other small group instructional modalities.

While this study found no differences in scores received based on demographics and TBL team point totals, future investigations should further evaluate whether biases exist by the individual completing the peer evaluation as this may have been mitigated by aggregate data. Furthermore, future research, specifically through a qualitative framework, will investigate student perceptions of the PET and process of peer evaluation for evidence of consequential validity (AERA 2014). Consequential validity seeks to explore the social consequences, both potential and actual, of receiving a score from an instrument and places the responsibility of the consequences on the instrument developers (Iliescu and Greiff 2021). The literature demonstrates wide variability in student perceptions of peer evaluation, where some students describe the process as motivating, but others have a degree of distrust in their peers’ ability to reliably evaluate them (Planas Llado et al., 2014). However, few have evaluated the consequences of peer evaluation on individual students in terms of their internalization of the evaluation, how it impacts their approach to TBL, and how the process may impact team dynamics.

## Conclusion

This study demonstrates additional evidence of validity to support the use of the PET in a TBL course. It highlights the importance of the critical elements of TBL, preparedness and accountability, to team dynamics, and these results further suggest that Prep scores can predict preparation for iRATs. Therefore, these results suggest that students can adequately evaluate their peers’ preparedness for TBL using the PET. Importantly, there were no differences in PET scores received across race/ethnicity and gender. Taken together, these findings further build validity evidence and start the exploration of possible forms of bias for this PET.

## Supplementary Information

Below is the link to the electronic supplementary material.

Supplementary Material 1

## Data Availability

The data that support the findings of this study are available on request from the corresponding author. The data are not publicly available due to privacy or ethical restrictions.
