# Supplementary material for: Assessing Validity and Bias in a Peer Evaluation Tool: Associations with Demographics and Performance in Team-based Learning
Source: Med Sci Educ. 2026 May 20;36(5):2183–90. doi: 10.1007/s40670-026-02759-8 (PMC13723551; doi:10.1007/s40670-026-02759-8)
Supplement: Supplementary file 1 [file 40670_2026_2759_MOESM1_ESM.docx]

**Table S1.** Study sample characteristics and distribution across cohorts.

|  | **N (%)** | **Cohort** | | | | |
| --- | --- | --- | --- | --- | --- | --- |
|  | All | 2020 | 2021 | 2022 | 2023 | 2024 |
| **All** | 205 | 32 (15.6%) | 48 (23.4%) | 39 (19%) | 40 (19.5%) | 46 (22.5%) |
| **Gender** |  |  |  |  |  |  |
| Woman | 135 (66%) | 26 (81.25%) | 29 (60.42%) | 24 (61.5%) | 28 (70%) | 28 (60.9%) |
| Man | 70 (34%) | 6 (18.75) | 19 (39.58%) | 15 (38.5%) | 12 (30%) | 18 (39.1%) |
| **Ethnicity** |  |  |  |  |  |  |
| Asian | 38 (18.6%) | 3 (9.4%) | 8 (16.65%) | 12 (30.8%) | 8 (20%) | 7 (15.2%) |
| Black/African American | 54 (26.5%) | 14 (43.75%) | 12 (25%) | 2 (5.1%) | 14 (35%) | 12 (26.1%) |
| Hispanic | 21 (10.3%) | 6 (18.75%) | 3 (6.25%) | 3 (7.7%) | 3 (7.5%) | 6 (13%) |
| Native American | 1 (0.01%) | 1 (3.1%) | - | - | - | - |
| White | 91 (44.6%) | 8 (25%) | 25 (52.1%) | 22 (56.4%) | 15 (37.5%) | 21 (45.7%) |
